# Novel Small Multilamellar Liposomes Containing Large Quantities of Peptide Nucleic Acid Selectively Kill Breast Cancer Cells

**DOI:** 10.3390/cancers14194806

**Published:** 2022-09-30

**Authors:** Galina Proshkina, Elena Shramova, Anastasiya Ryabova, Liat Katrivas, Clelia Giannini, Daniele Malpicci, Yael Levi-Kalisman, Sergey Deyev, Alexander Kotlyar

**Affiliations:** 1 Shemyakin-Ovchinnikov Institute of Bioorganic Chemistry, Russian Academy of Sciences, Miklukho-Maklaya St., 16/10, 117997 Moscow, Russia; 2 Prokhorov General Physics Institute, Russian Academy of Sciences, 38 Vavilova St., 119991 Moscow, Russia; 3 Department of Biochemistry and Molecular Biology, George S. Wise Faculty of Life Sciences and the Center of Nanoscience and Nanotechnology, Tel Aviv University, Ramat Aviv, Tel Aviv 69978, Israel; 4 Department of Chemistry, University of Milan, Via Golgi 19, 20133 Milan, Italy; 5 Institute for Life Sciences and the Center for Nanoscience and Nanotechnology, The Hebrew University of Jerusalem, Jerusalem 91904, Israel

**Keywords:** peptide nucleic acid, DARPins, liposomes, HER2, targeting delivery

## Abstract

**Simple Summary:**

We present, for the first time, the preparation of small (60–90 nm in diameter) liposomes containing extremely large amounts (~8000 molecules per vesicle) of short, cytosine-rich peptide nucleic acid. The outer surface of liposomes wasfunctionalized with scaffold molecules specific to tumor-associated antigen overexpressing in breast cancer. We have shown that targeted liposomesspecifically interact with cancer cells and reduce their viability in sub-nanomolar concentrations. The results presented here can be widely used in cancer therapy based on cytosine-rich PNA oligonucleotides.

**Abstract:**

Peptide nucleic acid (PNA) may be used in various biomedical applications; however, these are currently limited, due to its low solubility in aqueous solutions. In this study, a methodology to overcome this limitation is demonstrated, as well as the effect of PNA on cell viability. We show that extruding a mixture of natural phospholipids and short (6–22 bases), cytosine-rich PNA through a 100 nm pore size membrane under mild acidic conditions resulted in the formation of small (60–90 nm in diameter) multilamellar vesicles (SMVs) comprising several (3–5) concentric lipid membranes. The PNA molecules, being positively charged under acidic conditions (due to protonation of cytosine bases in the sequence), bind electrostatically to negatively charged phospholipid membranes. The large membrane surface area allowed the encapsulation of thousands of PNA molecules in the vesicle. SMVs were conjugated with the designed ankyrin repeat protein (DARPin_9-29), which interacts with human epidermal growth factor receptor 2 (HER2), overexpressed in human breast cancer. The conjugate was shown to enter HER2-overexpressing cells by receptor-mediated endocytosis. PNA molecules, released from lysosomes, aggregate in the cytoplasm into micron-sized particles, which interfere with normal cell functioning, causing cell death. The ability of DARPin-functionalized SMVs to specifically deliver large quantities of PNA to cancer cells opens a new promising avenue for cancer therapy.

## 1. Introduction

Peptide nucleic acid (PNA) is a DNA (RNA) analogue in which the nucleic base units are connected to each other by peptide bonds. In contrast to natural nucleic acids, PNA is uncharged and hydrophobic at physiological pH. The nucleic acid binds sequence-specifically to DNA or RNA via Watson–Crick hydrogen bonds between the nucleic bases. The stability of PNA-DNA and PNA-RNA hybrids is significantly higher than that of corresponding double-stranded (ds)DNA or DNA-RNA molecules [1,2] due to the lack of repulsion between PNA and DNA (RNA) strands in the hybrid. The sequence-specific binding to DNA and RNA, resistance to digestion by nucleases and proteases, and extremely high thermodynamic stability of PNA-DNA and PNA-RNA hybrids make PNA a promising candidate for a wide range of applications, including the sensing of DNA and RNA [3], molecular diagnostics, gene targeting and editing [4], MicroRNA targeting [5,6], antisense therapeutics, and others [7,8,9,10]. Certain PNA sequences possess antimicrobial and antiviral activities, and can be potentially used for the treatment of bacterial and viral diseases [11,12,13]. Their application in biomedicine is, however, limited by the relatively low solubility of PNA in aqueous solutions [14]. Various attempts have been undertaken to enhance the solubility of the nucleic acid at physiological conditions. Among them are the modification of the PNA backbone, the inserting of charged amino acid residues into the oligonucleotides, and the conjugation of hydrophilic molecules and polymers to PNA. All of these modifications, however, were achieved at the expense of the specificity of PNA interaction with DNA (RNA). Another way to address the challenge of limited solubility is to deliver PNA molecules to cells and tissues inside cargo vesicles. Liposomes are the most common and clinically established vesicles for the delivery of various bio-active compounds, such as hormones, peptides, proteins, DNA, and RNA, to cells, tissues, and organs (for reviews, see [15,16,17,18]). Several studies reported the encapsulation of PNA into liposomes at neutral pH [19,20,21]. The reported PNA-containing liposomes have been internalized into human erythroleukemic cells, causing PNA-mediated reduction of microRNA (miR-210) levels in the cytoplasm [20]. As far as we know, no data have been reported so far on the targeted delivery of PNA-encapsulated liposomes to cancer cells, and on their effect on cell viability. In order to enable specific delivery to pathological cells, liposomes should be functionalized with ligands capable ofselectively targeting receptors overexpressed on the plasma membrane. This can be performed bythe covalent attachment of antibodies, or genetically engineered antibody mimetic proteins to the liposome membrane (for reviews, see [15,16,17,22,23]).

Designed ankyrin repeat proteins (DARPins) are a sort of antibody mimetic proteins built on naturally occurring ankyrin repeats [24]. DARPins are small (13–20 kDa), possess excellent solubility in water, lack cysteine residues, are very stable, and are characterized by very high affinity to their protein targets [24,25,26,27]. A number of DARPin molecules that target human epidermal growth factor receptor 2 (HER2) [25,26,27,28], overexpressed in breast cancer and ovarian cells, have been synthesized.

We have recently reported a method for the preparation of small, unilamellar liposomes containing large quantities (thousands) of protein molecules per liposome [29]. The encapsulation was achieved through electrostatic interaction between negatively charged liposome membrane and positively charged proteins (at pH lower than pI). The proteoliposomes were functionalized with DARPin_9-29, which targets human epidermal growth factor receptor 2 (HER2), and were shown to be specifically internalized by HER2-overexpressing cells.

In this work, we report the preparation of small (60–90 nm in diameter), DARPin_9-29-functionalized liposomes containing extremely large amounts (~8000 molecules per vesicle) of short cytosine-rich PNA. The method is based on electrostatic binding of positively charged (at pH lower than 4) C-rich PNA molecules to negatively charged phospholipid membranes. The extrusion of the mixture through a 100 nm-pore size cellulose membrane yields relatively small multilamellar liposomes. These liposomes are composed of several spheres—one inside the other—and have much greater surface area compared to the unicameral vesicles of the same size. The great surface area enabled us to encapsulate as much as thousands of PNA molecules in a liposome.The liposomes were shown to specifically enter HER2-overexpressing breast cancer cells, and to strongly reduce their viability in sub-nanomolar concentrations.

## 2. Material and Methods

L-αPhosphatidylcholine (Avanti Polar Lipids, Soy 40%, 341602), composed mainly of ~40% phosphatidylcholine, ~16% phosphatidylethanolamine, and ~11% phosphatidylinositol, was used. All other reagents and chemicals (unless otherwise stated) were obtained from Sigma-Aldrich (St. Louis, MO, USA).

### 2.1. Preparation of DARPin-9_29

The protein expression and purification were described in an earlier study [29]. In short, the E. coli BL21(DE3) strain cells were transformed with the plasmid, pDARP. E. coli transformants were grown in auto-induction medium, TBP-5052 [30], containing 0.1 g/L ampicillin at 25 °C with vigorous aeration to OD of 12–14 at 600 nm. The cells were harvested by centrifugation at 6000× *g* for 10 min at 4 °C. The pellet was resuspended in a lysing buffer (20 mM Na-Pi, pH 7.5, 500 mM NaCl, 50 μg/mL lysozyme), and sonicated on a VibraCell (Sonics, Newtown, CT, USA) ultrasonic disintegrator 30 times for 10 s with 10 s intervals. The cellular debris was removed by centrifugation at 15,000× *g* for 20 min at 4 °C. The clear lysate containing his-tagged DARPin_9-29 was passed through a 0.22-μm filter and loaded onto a Ni2+ -NTA (GE Healthcare, Chicago, IL, USA) column equilibrated with: 20 mM Na-Pi, pH 7.5, 500 mM NaCl, and 30 mM imidazole. The protein elution was performed in accordance with manufacturer instructions. The yield of the purified protein was 84 mg per L of the culture.

### 2.2. PNA Synthesis and Characterization

The PNA oligomers, Flu-CCCTAA, Flu-CCCTAACCCTAA, Flu-CCCTAACCCTAACCCTAACCCT, Flu-TCACAC, Flu-TCTCACTCACAA, and Flu TCTCACTCACACTCTCACTCAC, were prepared by solid-phase peptide synthesis SPPS using fluorenylmethoxycarbonyl/benzhydryloxycarbonylFmoc/Bhoc protected PNA monomers (ASM Research Chemicals, Hannover, Germany) and a Rink resin carrying the 4-methylbenzhydrylamine hydrochloride salt group (Merck KGaANovabiochem, Darmstadt, Germany). The resin was downloaded to 0.2 mmoL/g with the first monomer of the sequence (C-end). Polypropylene syringes (10 mL) were used as reaction vessels. Semi-automatic SPPS was accomplished through the Biotage^®^ Initiator™ synthesizer, assisted by microwave irradiation [31] (see the detailed procedure of PNA synthesis and characterization in Supplementary Data).

### 2.3. Preparation of PNA-Encapsulated Liposomes

The phospholipid suspension was prepared as described previously [29]. Several grains of one of the following fluorescein-labelledPNA oligonucleotide sequences, CCCTAA, CCCTAACCCTAA, TCACAC, CCCTAACCCTAACCCTAACCCT, TCTCACTCACAA, and TCACACTCACACTCACACTCAC, were dissolved in ~200 µL of double-deionized water. The oligonucleotide concentration was calculated on the bases of fluorescein absorption (ε = 75.000 at 495 nm). The samples (~0.6 mL), containing 10 mM Na-acetate (pH 3.5), 1 mg/mL phospholipids, and 0.2 mM of one of the above PNA oligonucleotides, were frozen and thawed 5 times prior to extrusion (19 times) through a 100-nm pore size polycarbonate membrane using an Avanti Mini Extruder at ambient temperature. A volume of 50 µL of 1 M K-P_i_ (pH 12) was added to the suspension, and the sample was chromatographed on a Sepharose CL-2B column (10 × 35 mm) equilibrated with 20 mM K-P_i_ (pH 11.5). At a pH above 11, the above PNA oligonucleotides become negatively charged (due to the deprotonation of T-bases; pK = 9.5) and detach from the outer surface of the liposome. The liposome fraction, eluted in the void volume of the column, was collected and stored at 4 °C until used.

### 2.4. Covalent Coupling of DARPin_9-29 to Liposomes

DARPin_9-29 was covalently attached to the outer surface of the liposome membrane through a flexible ~17 angstroms spacer arm as described [29]. The excess of DARPin was separated from the liposomes using size-exclusion chromatography [29]. The void volume fraction contained DARPin_9-29-functionalized PNA-containing SMVs, or DARPin-PNA-SMVs were collected and stored at 4 °C until used.

### 2.5. Cell Cultures

The cell lines, SK-BR-3 (human breast adenocarcinoma, ATCC number HTB-30), MDA-MB-231 (human breast adenocarcinoma, ATCC number HTB-26), SK-OV-3 (human ovarian adenocarcinoma, ATCC number HTB-77), and HEK-293 (human embryonic kidney epithelial cells, ATCC number CRL-1573), were cultured inRPMI-1640 growth medium supplemented with 10% FBS, 2 μM L-glutamine, 100 μg/mL streptomycin, and 100 U/mL penicillin at 37 °C in a 5% CO_2_humidified atmosphere [32].

### 2.6. Cell Viability Assay

To study the in vitro cell cytotoxicity of DARPin-PNA-SMVs and PNA-SMVs, DARPin or PNA cells (~3 × 10^4^ per well) were inoculated onto 96-well plates (Corning, Glendale, AZ, USA) and grown overnight. After 24 h, the growth medium was replaced with the medium containing different concentrations of testing compounds (0–500 nM for DARPin-PNA-SMVs or PNA-SMVs, and 0–1000 nM for DARPin or PNA), and the experiment was carried out for 72 h. Cell viability was determined by MTT assay [33]. In short, the medium was removed, and 100 μL of 0.5 g/L MTT solution (tetrazolium dye, 3-(4,5-dimethyl-2-thiazolyl)-2,5-diphenyl-2H-tetrazolium bromide) in serum-free medium was added to the wells; the incubation was for 1 h at 37 °C in a 5% CO_2_atmosphere. The MTT solution was removed, and 100 μL of DMSO (dimethyl sulfoxide) was added to each well to dissolve formazan crystals. The optical density was measured using the Infinite M100 Pro microplate reader (Tecan, Grödig, Austria) at 570 nm. The relative viability was calculated as the ratio (in percent) of the average optical density in wells with treated cells to that in wells with the untreated ones (control).

### 2.7. Confocal Microscopy

SK-BR-3 and MDA-MB-231 cells were cultured on glass bottom dishes (WillCo, Amsterdam, Holland) overnight in 5% CO_2_ at 37 °C. The binding and internalization of DARPin-PNA-SMVs to SK-BR-3 and MDA-MB-231 cells was measured as follows. The cells were incubated in a growth medium containing 50 nMDARPin-PNA-SMVs for 30 min at 37 °C. The cells were washed twice with the medium and subsequently incubated in the fresh one for 6, 24, and 48 h at 37 °C. The nuclei were stained with Hoechst 33342 as described in [32].DARPin-PNA-SMVs containing the fluorescein-labelled PNA were excited at 488 nm; the emission was recorded between 500 and 550 nm. Hoechst was exited at 700 nm (two-photon excitation) using a femtosecond laser. The Hoechst emission was detected between 400 and 600 nm. The 63× oil Plan-Apochromat objective with a numerical aperture of 1.4 was used to obtain high-quality images.

### 2.8. Hypoploid DNA Determination

The hypoploid DNA fraction was estimated as described in [34]. In short, SK-BR-3 cells were inoculated in a 12-well plate (10^5^ cells per well) in the complete growth medium. On the next day, DARP-PNA-SMVs (500 nM) wereadded. After 36 h, the cells were removed from the wells using Versen solution, and centrifuged for 10 min at 1000× *g* at 4 °C. The pellets were suspended in phosphate-buffered saline and centrifuged as above. Each pellet was suspended in 1 mL of 70% ethanol and left on ice for 30 min. Then, the cells were centrifuged for 5 min at 1000× *g* at 4 °C. The supernatant was removed, and the cells were suspended in 200 μL of phosphate-buffered saline containing 50 μg/mL propidium iodide and 0.5 μg/mL RNase A (QIAGEN, Hilden, Germany). After incubation for 15 min at ambient temperature, the samples were analyzed on the NovoCyte3000 (Bioscience, Allentown, PA, USA) flow cytometer. Propidium iodide was excited at 561 nm and detected using a 615/20 bandpass filter.Staurosporin (5 μM) was used as a positive apoptotic control. The necrotized cells were prepared by a 10-min treatment of SK-BR-3 cells at 75 °C.

### 2.9. Competition Experiment and Estimation of HER2-Level in Cells

HER2-positive SK-BR-3 cells were incubated for 10 min at 37 °C in the presence of 50 nM of FITC-labeled DARPin, DARPin-PNA-SMVs, or DARPin. Then, the cells were washed twice with PBS and analysed using a NovoCyte 3000 Flow Cytometer System (ACEA Biosciences Inc., San Diedo, CA, USA). The excitation was at 488 nm, and the emission was detected using 530 ± 30 nm band pass filter. The data were analyzed using NovoExpress software.

### 2.10. Cryo-TEM Measurements

The experiment was performed on an FEI Tecnai 12 G^2^ Spirit TWIN TEM as described in [29]. A 3μLdrop of the sample (liposomes containing CCCTAA) was applied to a glow-discharged TEM grid (300-mesh Cu grid) coated with a holey carbon film (Lacey substrate, Ted Pella, Ltd., Redding, CA, USA). The excess liquid was blotted, and the specimen was vitrified by a rapid plunging into liquid ethane pre-cooled with liquid nitrogen using Vitrobot Mark IV (FEI). The vitrified samples were then transferred to a cryo-holder (Gatan model 626) and examined at −177 °C using a FEI Tecnai 12 G^2^Spirit TWIN TEM. The images were recorded by a 4K × 4K (FEI Eagle) CCD camera at 120 kV in low-dose mode. TIA (Tecnai Imaging & Analysis, Weinheim, Germany) software was used to record the images.

## 3. Results

### 3.1. Encapsulation of PNA in Liposomes

**Figure 1:**
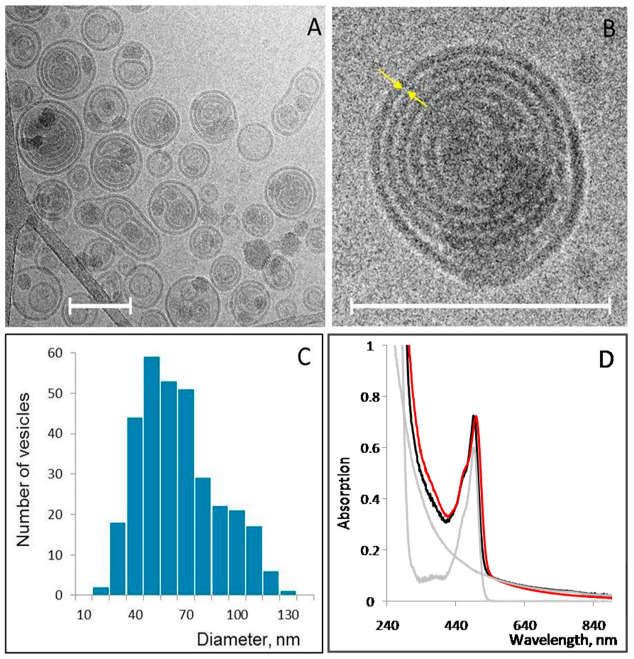
Cryo-TEM images of liposomes containing CCCTAA (**A**,**B**), statistical size analysis of more than 300 liposomes (**C**), and absorption spectroscopy analysis of PNA-SMVs (**D**). (**A**,**B**) The average diameter of the liposomes equals 64 nm. Scale bars = 100 nm. The liposomes containing CCCTAA were prepared as described in Materials and Methods. (**D**) Liposomes were prepared as described in Materials and Methods. The spectra ofSMVs loaded with fluorescein-labeled (a fluorescein residue is attached to the N-end the oligonucleotide) CCCTAA—orange curve; 1 mg/mL bear (not loaded with PNA) liposomes (light-scattering spectrum); and 8.6 µM Flu-CCCTAA (the extinction coefficient of fluorescein at 495 nm is equal to ~70 mM^−1^cm^−1^)—grey curves. The sum of spectral curves corresponding to fluorescein and liposomes—black curve fits well with the experimental spectrum (red curve). The molar concentration of bear liposomes in 1 mg/mL suspension is equal to 1.1 nM [29]. The PNA-to-liposome molar ratio, which corresponds to the number of the PNA molecules per liposome, is, thus, equal to (8600 nM/1.1 nM) ~8000.

At neutral pH, the PNA oligonucleotides used here, CCCTAA, TCACAC, CCCTAACCCTAA, TCTCACTCACAA, CCCTAACCCTAACCCTAACCCT, and TCTCACTCACACTCTCACTCAC, are almost insoluble.The solubility of the oligonucleotides is, however, strongly increased upon lowering the pH (Figure S1). At pH lower than 5, cytosine bases (pK = 4.5) composing the oligonucleotides undergo protonation and become positively charged. The positively charged (at mild acidic pH values) oligonucleotides are capable of binding electrostatically to negatively charged membranes made of natural phospholipids. Extrusion of the PNA-phospholipid mixtures through a 100 nm-pore size filter yielded relatively small (average diameter of about 64 nm; Figure 1C) vesicles. Cryo-TEM images show that the majority of the vesicles are onion-shaped and composed of several (3–5) concentric lipid layers (Figure 1A); in some liposomes, small multilamellar vesicles are encapsulated in bigger ones. We will term these lipid vesicles as SMVs (small multilamellar vesicle) throughout the paper. The gap between the concentric spheres in the structure, in many cases, does not exceed the width of the bilayer (Figure 1B, see yellow arrows). The average distance between the spheres calculated from the images (~60 measurements) is equal to 2.8 ± 2 nm. Similar structures were seen in SMV samples, which contained different PNA oligonucleotides (see above).

The absorption spectrum of the PNA-containing liposomes, PNA-SMVs, is presented in Figure 1D (orange curve). The absorption peak with a maximum at 495 nm corresponds to fluorescein (Flu) covalently attached to the N-terminus of the oligonucleotide. The spectral analysis (Figure 1D) showed that as many as 8000 PNA molecules can be carried by a liposome. This extraordinary high PNA binding capacity is due to the very large membrane surface area of the lipid vesicles. As seen in Figure 1, the membranes are densely packed inside the vesicle (Figure 1B). The PNA molecules can bind to both sides of each of the concentric membranes composing the SMVs, leading to the observed high level of encapsulation.

### 3.2. Specific Internalization of DARPin-PNA-SMVs into SK-BR-3

**Figure 2:**
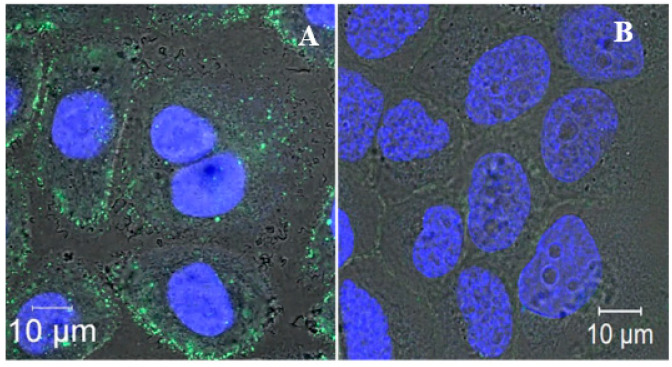
Specific internalization of DARPin-PNA-SMVs by HER2-overexpressing cells. SK-BR-3 (**A**) and MDA-MB-231 (**B**) cells were incubated with 50 nMDARPin-PNA-SMVs, containing the fluorescein-labelled oligonucleotide (Flu-CCCTAA) for 30 min at 37 °C. The cells were washed twice with culturing medium and subsequently incubated at 37 °C for 6 h (see Materials and Methods). Nuclei were stained with Hoechst 33342. Superimposed confocal fluorescent images of the cells in blue-green and blue-red fluorescence channels are presented.

DARPin_9-29, which is known to interact selectively and specifically with HER2 receptors overexpressed in some types of cancers, was covalently conjugated to the outer surface of the liposomes, as described in detail in Materials and Methods, and in [29]. The conjugate, DARPin-PNA-SMVs, containing the encapsulated fluorescein-labelled PNA, wereincubated with HER2-overexpressing SK-BR-3 (~10^6^ molecules per cell) [35] and MDA-MB-231 cells expressing normal amounts of HER2 (~10^4^ molecules per cell) for epithelial cells [35] for 30 min at 37 °C. Treatment with the liposomes led to the appearance of bright green fluorescent spots, corresponding to the PNA, in the cytoplasm next to the membrane of SK- BR-3 cells (Figure 2A),whereasno such spots were detected in MDA-MB-231 cells treated under identical conditions (Figure 2B).

**Figure 3:**
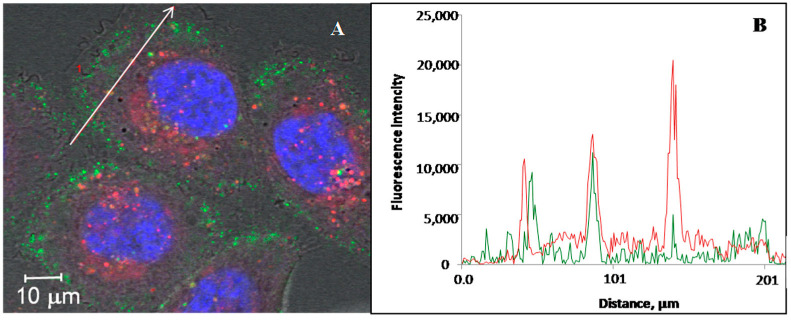
Internalization of DARPin-PNA-SMVs by SK-BR-3 cells. (**A**) The cells were incubated with 50 nMDARPin-PNA-SMVs, containing the fluorescein-labelled CCCTAA oligonucleotide, for 30 min at 37 °C (see Materials and Methods). The cells were washed twice with culturing medium and subsequently incubated at 37 °C for 6 h (see Materials and Methods). The cells were further treated with Hoechst 33342 and LysoTracker to stain the nucleus and lysosomes, respectively. Fluorescence intensity profiles along the white arrow areshown in panel. (**B**) The green curve in B corresponds to the fluorescence intensity profiles of DARPin-PNA-SMVs, and red curves to the fluorescence intensity profiles of the lysosome-specific dye.

The co-staining of DARPin-PNA-SMVs with a lysosomal marker (Figure 3) showed that the PNA is localized mainly in the lysosomes, supporting the endocytosis-mediated mechanism of the liposome entry.

**Figure 4:**
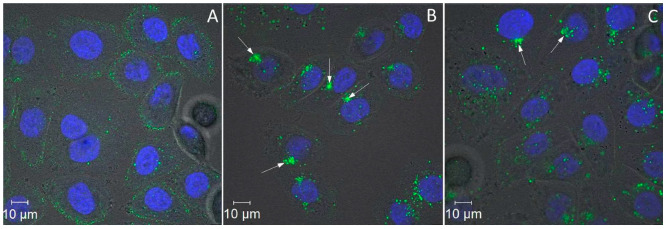
Time-course of the internalization process. SK-BR-3 cells were incubated with 50 nMDARPin-functionalized SMVs, containing the fluorescein-labelled CCCTAA, for 30 min at 37 °C (see Materials and Methods). The cells were washed twice with the culturing medium and subsequently incubated at 37 °C for 6 (**A**), 24 (**B**), and 48 (**C**) hours. Large colored areas pointed by arrow correspond to PNA aggregates. The cells were further treated with Hoechst 33342 to stain the nucleus.

The localization of the internalized dye was followed by fluorescent confocal microscopy for two days. After incubation for 6 h at 37 °C, the dye is located in the cytoplasm next to the cell membrane (Figure 4A); small bright spots seen on the image are associated with SMVs loaded with the fluorescein-labelled PNA. After incubation for 24 h, most of the PNA is located near the nucleus (Figure 4B); large colored areas are seen in the image (pointed by arrows in Figure 4B), along with smaller ones.The PNA distribution did not change considerably on the next day (48 h after the process began). Large bright areas (pointed by arrows in Figure 4C) can still be seen in the image.

### 3.3. The Effect of DARPin-PNA-SMVs on Viability of SK-BR-3 Cells

**Figure 5:**
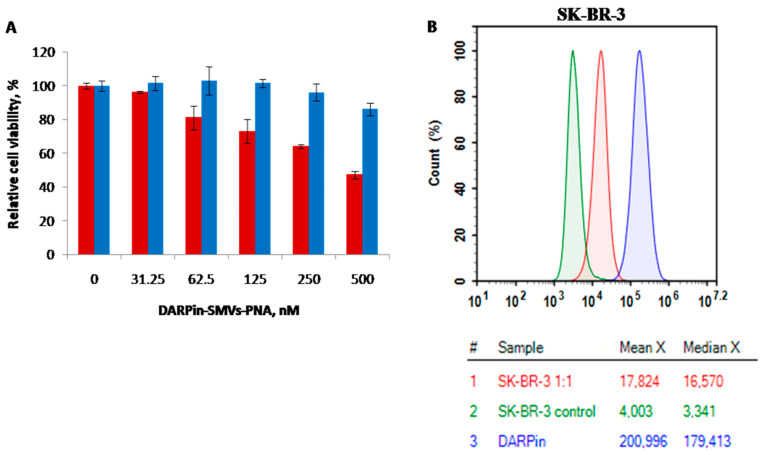
DARPin-PNA-SMVs’ interaction with HER2-positive cells. (**A**) The effect of DARPin- PNA-SMVs on cell viability. SK-BR-3 (red columns) and MDA-MB-231 cells (blue columns) were incubated in the presence of DARPin-PNA-SMVs (concentrations are shown on the horizontal axis) for 72 h. Relative cell viability was measured after 72 h, as described in Materials and Methods. Error bars correspond to standard deviation of threeindependent experiments. (**B**) Interaction of DARPin-PNA-SMVs with HER2 receptor in the absence (blue line) or presence (red line) of added DARPin. The green line corresponds to autofluorescence of non-treated SK-BR-3 cells. The data were obtained with flow cytometry, as described in Materials and Methods.

SK-BR-3 cells overexpressing HER2 and MDA-MB-231 cells expressing normal amounts of HER2 were incubated with nanomolar concentrations of DARPin-PNA-SMVs for 72 h at 37 °C. As evident from Figure 5A, the viability of only HER2-overexpressing SK-BR-3 cells was affected by the treatment; MDA-MB-231 cells expressing normal amounts of the receptor treated under identical conditions retained high viability. The liposome conjugates containing different C-rich PNA sequences, CCCTAACCCTAA, CCCTAACCCTAACCCTAACCCT, TCACAC, TCTCACTCACAA, and TCTCACTCACACTCTCACTCAC, produced similar effects on cell viability (Figure S2).

We have demonstrated that PNA, DARPin, and non-functionalized SMVs-PNA almost do not affect the cells (Figure S3). Slight (no more than 3–4%) inhibition by non-functionalized liposomes (Figure S3, left panel) is probably the result of non-specific endocytosis of the liposomes [36]. We have also shown that DARPin-SMVs-PNA do not affect the viability of normal epithelial cells (Figure S4).

We have demonstrated that treatment with the PNA-carrying SMVs induces apoptosis in the cells (Figure S5). As seen in Figure S5, incubation with DARPin-PNA-SMVs leads to strong fragmentation of the DNA in SK-BR-3 cells, which is consistent with an apoptotic mechanism of cell death [37,38].

In order to confirm that the binding of DARPin-functionalized liposomes occurs through the interaction of DARPin with HER2 receptors on the cell surface, we investigated the effect of free DARPin on the liposomes (DARPin-PNA-SMVs) binding to HER2-positive cells. The addition ofDARPinstrongly reduced the median fluorescence of DARPin-SMVs-PNA (Figure 5B).The results of these competitive flow cytometry experiments (Figure 5B) clearly show that DARPin is involved in the binding of DARPin-PNA-SMVs to the cells.

In order to prove that HER2 plays a crucial role in liposome-induced cell death, we also tested the influence of DARPin-PNA-SMVs on the viability of HER2-negative SK-OV-3 ovarian carcinoma cells. SK-OV-3 can overexpress HER2 [35], but shedding of the extracellular part of HER2 [39] downregulates the receptor. We have demonstrated by flow cytometry (Figure S6) that SK-OV-3 doesnot express the functionally active HER2 receptor; the mean florescence of the cells treated with FITC-labelled DARPin is equal to that in the untreated cells, whereasfor HER2-positive SK-BR-3 cells, the mean fluorescence is ~29 times higher in the treated cells (Figure S6). The lack of the effect of DARPin-PNA-SMVs on the viability of SK-OV-3 cells is then probably due to the inability of the cells to bind strongly to DARPin.

## 4. Discussion

We have discovered that extruding a mixture of natural phospholipids and cytosine-rich PNA through a 100 nm cellulose membrane at mild acidic pH yields relatively small (~60 nm in diameter) lipid vesicles composed of several concentric spheres. These novel multilamellar lipid vesicles, SMVs, are much smaller than large multilamellar vesicles (from 500 to 5000 nm in diameter) commonly used in biomedical research [40]. The SMVs can carry very large quantities of PNA: about 8000 PNA in the vesicle. The high binding capacity is due to the electrostatic interaction of PNA (positively charged at pH below 5) with the negatively charged membranes composing the vesicle.

**Figure 6:**
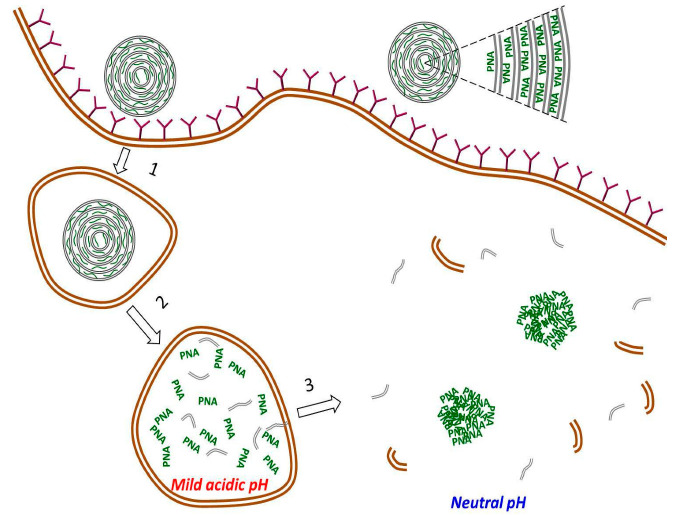
Scheme of the pathway leading to formation of PNA aggregates in the cytoplasm. The PNA molecules (short green curves) are bound to both sides of each of the concentric lipid membranes composing the vesicles (see the right-upper corner). These multilamellar vesicles, characterized by large membrane surface area per volume, are capable of binding large amounts of membrane-bound PNA oligonucleotides. Internalization of the vesicles into the cell includes the following steps: 1—binding of a DARPin-PNA-SMV to HER2 receptors on the surface of the HER2-overexpressing cell and encapsulation of the liposome in the endosome; 2—maturation of the endosome to lysosome and digestion of the lysosome-entrapped liposome; 3—release of PNA monomers along with digested membrane fragments from the acidic lumen (pH 4.5–5) to neutral cytoplasm (pH~7.4), followed by aggregation of PNA monomers into large PNA aggregates.

The initial idea of the work was to internalize large amounts of PNA complementary to the G-rich telomeric strand, and to study the effect of the nucleic acid on cell viability. We have demonstrated, however, that all tested sequences had a similar effect on cell viability (Figure S3), pointing out that the observed effect is not due to sequence-specific interactions of PNA with telomeres. At the same time, we have found that large (micron-sized) PNA granules are formed in the cytoplasm of DARPin-PNA-SMVs-treated cells (Figure 4). Based on the results of confocal fluorescent microscopy (Figure 4 and Figure S7), we proposed a mechanism of the granule formation in the cell (Figure 6), which includes the following steps: 1—internalization of large quantities of C-rich PNA oligonucleotides entrapped inside DARPin-functionalized SMVs into HER2-overexpressing cells; 2—digestion of the liposome by lysosomal enzymes followed by the release of the PNA oligonucleotides to the lumen; 3—release of the indigestible PNA from a matured lysosome to the cytoplasm. The C-rich PNA molecules are highly soluble at pH below 5, and are almost insoluble at neutral pH (Figure S2). Thus, the aggregation triggered by the pH increase from 4.5–5 (pH in the lysosome lumen) to 7.4 (pH in the cytoplasm) takes place upon the release of the nucleic acid from the lysosomes. As seen in Figure 4 and Figure S4, insoluble micron-sized colored granules are formed in the cytoplasm of DARPin-PNA-SMVs-treated SK-BR-3 cells. We suggested that these granules interfere with normal cell functioning and reduce cell viability (Figure 6).

The results reported here can open new promising avenues for cancer therapy employing the ability of cytosine-rich PNA oligonucleotides, being specifically delivered to cancer cells, to form large aggregates in the cytoplasm, leading to cell death.

## 5. Conclusions

In conclusion, in this work, we present, for the first time, the preparation of small (60–90 nm in diameter) DARPin_9-29-functionalized liposomes containing extremely large amounts (~8000 molecules per vesicle) of short, cytosine-rich PNA. The liposomes were shown tospecificallyenter HER2-overexpressing breast cancer cells by receptor-mediated endocytosis, and to strongly reduce their viability in sub-nanomolar concentrations. To the best of our knowledge, this is the first work devoted to the targeted delivery of PNA-encapsulated liposomes to cancer cells.

The results presented here can be widely used in cancer therapy based on cytosine-rich PNA oligonucleotides.

## Data Availability

All data needed to evaluate the conclusions in the paper are present in the paper and Supplementary Materials.

## Supplementary Materials

The following supporting information can be downloaded at: https://www.mdpi.com/article/10.3390/cancers14194806/s1, Figure S1: Solubility of C-rich PNA oligonucleotides under mild acidic and neutral pH conditions; Figure S2: The effect of 6-, 12-, and 22-base PNA oligonucleotideson viability of SK-BR-3 cells; Figure S3: The effect of DARPin, PNA, and PNA- SMVs on viability of SK-BR-3 and MDA-MB-231 cells; Figure S4: The effect of DARPin-PNA SMVs on viability of normal epithelial and HER2-negative carcinoma cells; Figure S5: Hypoploid DNA determination; Figure S6: HER2-level estimation; Figure S7: Confocal microscopy of PNA granule formation in living cells. Methods: PNA synthesis and characterization.
