# Supplementary material for: Novel Small Multilamellar Liposomes Containing Large Quantities of Peptide Nucleic Acid Selectively Kill Breast Cancer Cells
Source: Cancers (Basel). 2022 Sep 30;14(19):4806. doi: 10.3390/cancers14194806 (PMC9564164; doi:10.3390/cancers14194806)
Supplement: Supplementary file 1 [file cancers-14-04806-s001.zip › cancers-1899272-supplementary.pdf]

Solubility of C-rich PNA oligonucleotides under mild acidic and neutral pH conditions

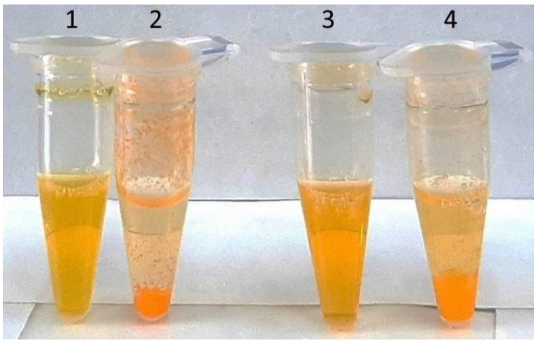

Figure S1. Solubility of C-rich PNA oligonucleotides under mild acidic and neutral pH conditions. Fluorescein-labeled PNA oligonucleotides, CCCTAA (1 and 2) and TCACAC (3 and 4) were incubated in 0.1 M Na-Acetate pH 4 (1 and 3) and 7 (2 and 4) for 30 min under ambient conditions. The image of the tubes was taken by a digital camera.

The effect of 6-, 12-, and 22-base PNA oligonucleotides on viability of SK-BR-3 cells

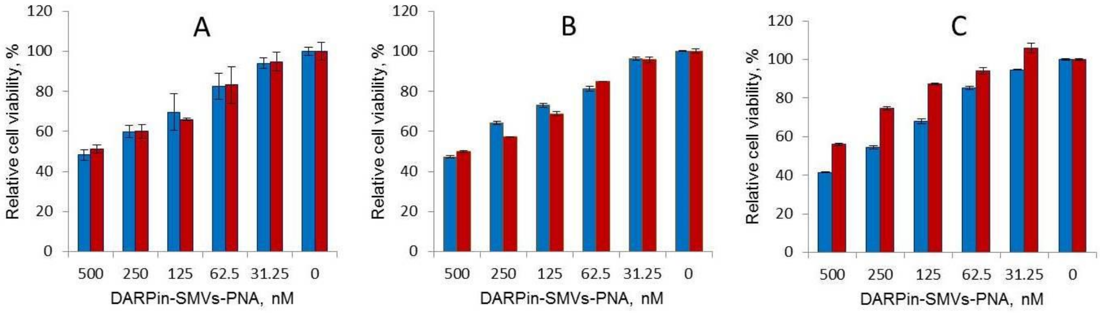

Figure S2. The effect of various fluorescein-labeled PNA oligonucleotides on viability of SK-BR-3 cells. A - CCCTAA (blue columns) and TCTCAC (red columns); B - CCCTAACCCTAA (blue columns) and TCTCACTCACAA (red columns); C - CCCTAACCCTAACCCTAACCCT (blue columns) and TCACACTCACACTCACACTCAC (red columns). The cells were incubated in the presence of DARPin-SMVs containing the above oligonucleotides at concentrations indicated on the horizontal axis as described in Materials and materials for 72 hours at 37°C. Relative cell viability was measured as described in Materials and methods.

The effect of DARPin, PNA and SMVs-PNA on viability of SK-BR-3 and MDA-MB-231 cells

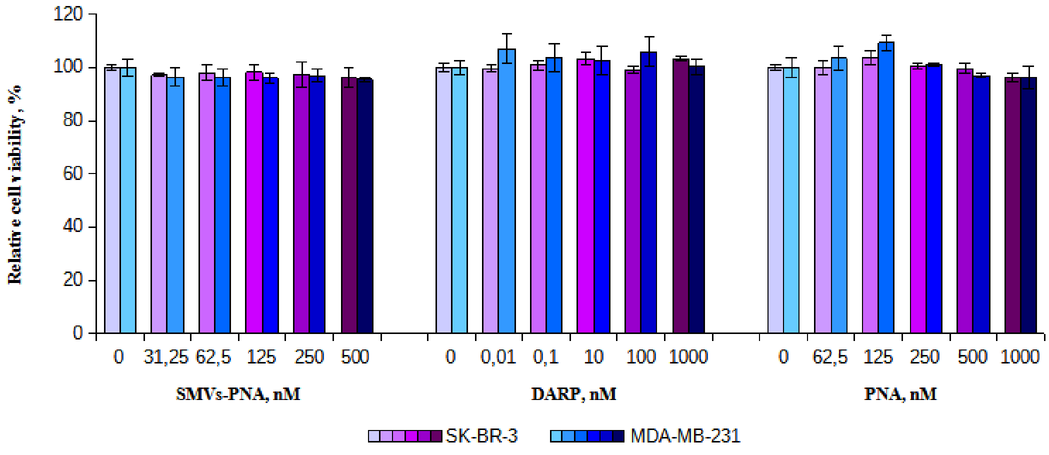

Figure S3. The effect of DARPin, PNA and SMVs-PNA on viability of SK-BR-3 and MDA-MB-231 cells. The cells were incubated in the presence of SMVs-PNA (liposomes loaded with (CCCTAA), PNA (CCCTAA) or DARPin at concentrations indicated on the horizontal axis as described in Materials and materials for 72 hours at 37°C. Relative cell viability was measured as described in Materials and methods.

The effect of DARPin-SMVs-PNA on viability of normal epithelial and HER2-negative carcinoma cells.

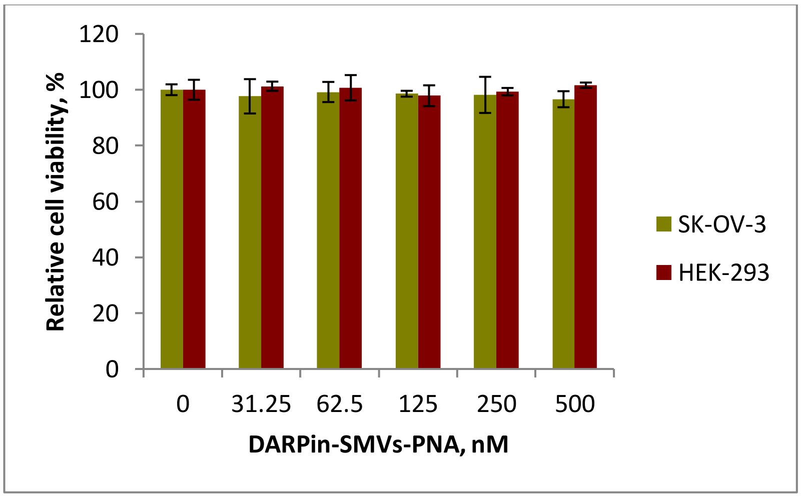

Fifure S4. The effect of DARPin-SMVs-PNA on viability of normal epithelial (HEK-293) and HER2-negative (SK-OV-3) carcinoma cells. The cells were incubated in the presence of DARPin-SMVs-PNA (CCCTAA) at concentrations indicated on the horizontal axis as described in Materials and materials for 72 hours at 37°C. Relative cell viability was measured with MTT assay as described in Materials and methods.

Hypoploid DNA determination

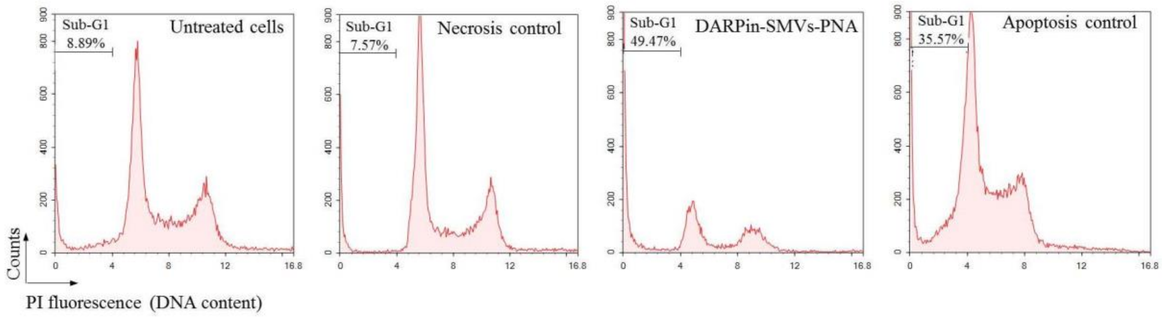

Figure S5. DNA fragmentation (Sub-G1 detection) was conducted by flow cytometry analysis of Propidium Iodide (PI)-stained cells as described in Materials and methods. SK-BR-3 cells were treated with 0.5 µM DARPin-SMV-PNA (liposomes loaded with CCCTAA). The cells treated with 2 mM $CaCl_2$ for 3 hours were used as a positive control for apoptosis. The necrotized cells were prepared by 10-min treatment of SK-BR-3 cells at 75°C. DNA fragmentation is shown (in percent) in the upper left corner of the panels.

HER2-level estimation

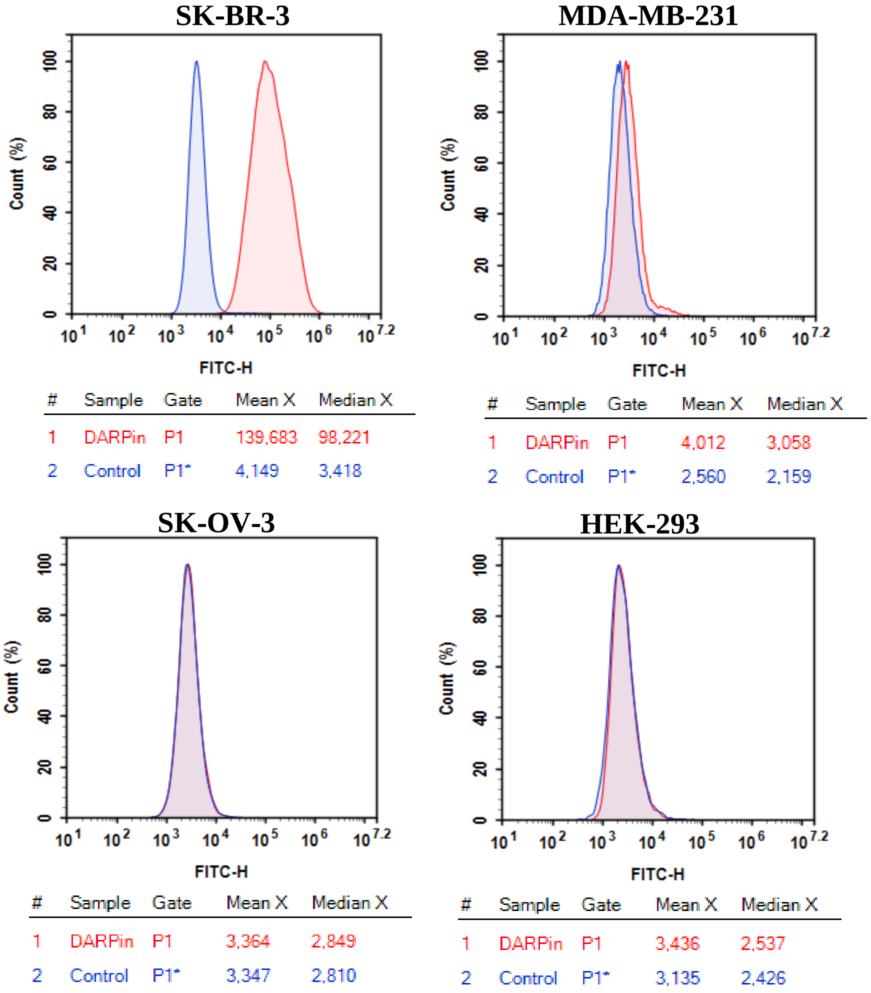

Figure S6. Estimation of HER2-level on cells by flow cytometry. SK-BR-3 (upper left pictogramm), MDA-MB-231 (upper right pictogramm), HEK-293T (bottom left pictogramm) and SK-OV-3 cells (bottom right pictogramm) were treated with 50 nM of FITC-labeled DARPin for 10 min at 37°C. The non-bound protein was subsequently washed with PBS and the cells were analized by flow cytometry. Blue line on each pictogramm corresponds to untreated cells (control of autofluorescence), red line corresponds to cells treated with DARPin.

Confocal microscopy of PNA granuls formation in living cells

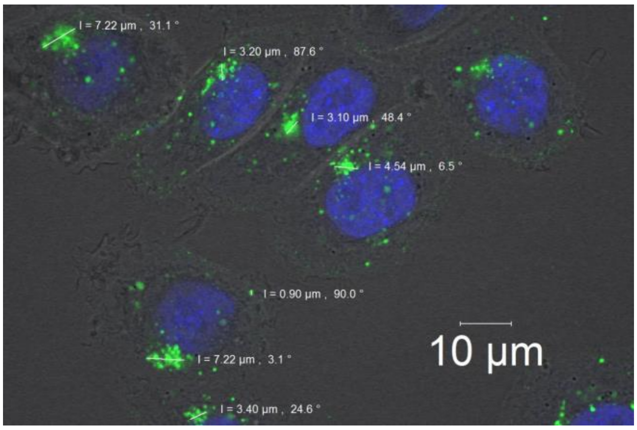

Figure S7. Formation of big PNA granuls in the cytoplasm. SK-BR-3 cells were incubated with 50 nM DARPin-PNA-SMVs, containing a fluorescein-labeled CCCTAA PNA sequence for 30 min at 37°C. The cells were washed twice with culturing medium and subsequently incubated at 37°C for 24 hours (see Materials and methods). Nuclei were stained with Hoechst 33342. Superimposed confocal fluorescent images of the cells in blue-green channels are presented. Sizes of the PNA aggregates (green areas) are shown.

PNA synthesis and characterization

The fluorescein-labelled (at the N-end) PNA oligomers: CCCTAA, TCACAC, CCCTAACCCTAA, TCTCACTCACAA, CCCTAACCCTAACCCTAACCCT and TCTCACTCACACTCTCACTCAC were prepared by solid phase peptide synthesis (SPPS) using Fluorenylmethoxycarbonyl/benzhydryloxycarbonyl Fmoc/Bhoc protected PNA monomers (ASM Research Chemicals) and a Rink resin carrying 4-methylbenzhydrylamine hydrochloride salt group (MBHA resin 0.74 mmoL/g, purchased from Merck KGaA Novabiochem). The semi-automatic SPPS was accomplished through the Biotage® Initiator™ synthesizer, assisted by microwave (MW) irradiation essentially as described in [Zanella et. Al, (2015). ChemistryOpen 4, 633– 641].

Polypropylene syringes (10 mL) were used as reaction vessels. The MBHA resin (130 mg, 0.74 mmoL/g,) was neutralized with 5% N,N-Diisopropylethylamine (DIPEA) in Dichloromethane (DCM) and downloaded to 0.2 mmoL/g with the proper first monomer (0.026 mmol). The unreacted amino groups were capped with a solution of Ac_2O/Pyridine/ dimethylformamide (DMF) 1:2:2. A deprotection steps were carried out adding 25% piperidine in DMF (3.0 mL), the reaction was performed at ambient temperature for 5 min. The resin was washed with DMF.

To a solution of the desired Fmoc-PNA-OH (3.0 eq with respect to the resin) in DMF (3.5 mL) Hexafluorophosphate Azabenzotriazole Tetramethyl Uronium, HATU (2.7 eq), N, N-Diisopropylethylamine, DIPEA (3.0 eq) and 2,6-Lutidine (4.5 eq) were added, and the resulting mixture was shaken for 2 min, the activated acid was then added to the resin in the reaction vessel of the synthesizer and the coupling reaction, assisted by microwaves, was carried out at 40 °C for 60 min. The beads were washed with DMF. The cycle (Fmoc deprotection and coupling) was repeated for each PNA monomer until the completion of the sequence.

A Fmoc-6-Ahx-OH linker was added with the same procedure at the N-end. A 5-fold excess of Fluorescein isothiocyanate (FITC) was then added, with DIPEA (8 eq) in DMF. The reaction was left on a shaker overnight a room temperature.

Cleavage of the resin was performed using a mixture of trifluoroacetic acid (TFA)/ trifluoromethanesulfonic acid (TFMSA)/thioanisole/m-cresol 6:2:1:1. The resin was washed with TFA and filtered off. The filtrate was concentrated and the PNA was precipitated adding Et_2O. The PNA oligomer (bright yellow solid) was washed several times with Et_2O and the dried in vacuo. Liquid chromatography–mass spectrometry (LC-MS) was used to confirm purity and identity of the synthesized oligonucleotides.

Mass spectra were recorded on a Thermo Fisher (Thermo Fisher Scientific, Waltham, MA USA) LCQ Fleet Ion Trap Mass Spectrometer equipped with UltiMate™ 3000 HPLC system, using an analytical Agilent ZORBAX Eclipse XDB-C18 column and a linear gradient of solvent A [Water (+0.1% formic acid)] and B [acetonitrile (+ 0.1 % formic acid)] starting from 5% B to 100% B in 60 minutes.

MALDI-TOF-MS spectra were recorded on the instrument Bruker Microflex™ LT, supporting the sample on α-cyano-4-hydroxycinnamic acid or sinapinic acid matrices. The sample was mixed in equal volumes with the matrix solution: a small amount (1 μL) of this mixture was spotted on the target surface. The target matrix was dried at room temperature and then analysed. Mass spectra of the synthesized oligonucleotides were recorded on a Thermo Fisher (Thermo Fisher Scientific, Waltham, MA USA) LCQ Fleet Ion Trap Mass Spectrometer equipped with UltiMate™ 3000 HPLC system, using an analytical C18 column (Agilent, USA). The oligonucleotides were eluted from the column in 0.1% formic acid with a linear acetonitrile gradient from 5 to 100%. MALDI-TOF-MS spectra of the oligonucleotides were recorded on Bruker Microflex™ LT. All oligonucleotides eluted from HPLC were chromatographically pure; the estimated masses of all synthesized PNA oligonucleotides correspond nicely with their chemical structures.
